# Exploring the Fecal Metabolome in Infants With Cow's Milk Allergy: The Distinct Impacts of Cow's Milk Protein Tolerance Acquisition and of Synbiotic Supplementation

**DOI:** 10.1002/mnfr.202400583

**Published:** 2024-12-12

**Authors:** Pingping Zhu, Mariyana V. Savova, Alida Kindt, the PRESTO study team, Harm Wopereis, Clara Belzer, Amy C. Harms, Thomas Hankemeier

**Affiliations:** ^1^ Metabolomics and Analytics Centre Leiden Academic Centre for Drug Research Leiden University Leiden The Netherlands; ^2^ Danone Research & Innovation Utrecht The Netherlands; ^3^ Laboratory of Microbiology Wageningen University Wageningen The Netherlands

**Keywords:** bifidobacteria, early life, food allergy, fructooligosaccharides, metabolomics

## Abstract

**Scope:**

Cow's milk allergy (CMA) is one of the most prevalent food allergies in early childhood, often treated via elimination diets including standard amino acid‐based formula or amino acid‐based formula supplemented with synbiotics (AAF or AAF‐S). This work aimed to assess the effect of cow's milk (CM) tolerance acquisition and synbiotic (inulin, oligofructose, *Bifidobacterium breve* M‐16 V) supplementation on the fecal metabolome in infants with IgE‐mediated CMA.

**Methods and Results:**

The CMA‐allergic infants received AAF or AAF‐S for a year during which fecal samples were collected. The samples were subjected to metabolomics analyses covering gut microbial metabolites including SCFAs, tryptophan metabolites, and bile acids (BAs). Longitudinal data analysis suggested amino acids, BAs, and branched SCFAs alterations in infants who outgrew CMA during the intervention. Synbiotic supplementation significantly modified the fecal metabolome after 6 months of intervention, including altered purine, BA, and unsaturated fatty acid levels, and increased metabolites of infant‐type *Bifidobacterium* species: indolelactic acid and 4‐hydroxyphenyllactic acid.

**Conclusion:**

This study offers no clear conclusion on the impact of CM‐tolerance acquisition on the fecal metabolome. However, our results show that 6 months of synbiotic supplementation successfully altered fecal metabolome and suggest induced bifidobacteria activity, which subsequently declined after 12 months of intervention.

## Graphical abstract

The effect of synbiotic supplementation on the fecal metabolome in infants with cow's milk allergy was assessed using a longitudinal metabolomics approach. Analysis of fecal samples revealed that synbiotic intake altered the purine, bile acid, and unsaturated fatty acids levels. Notably, the supplementation led to an increase in aromatic acid metabolites, suggesting successful colonization. No clinical link between these metabolome alterations and allergy outgrowth was made.

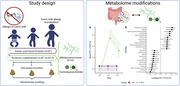

**Abbreviations**
AAF: amino acid‐based formula
AAF‐S: amino acid‐based formula with synbiotic
BSCFA: branched short‐chain fatty acid
BSH: bile salt hydrolase
CI: confidence interval
CM: cow's milk
CMA: cow's milk allergy
DBPCFC: double‐blind, placebo‐controlled food challenge
FOS: fructooligosaccharide(s)
GM: gut microbiome
HMO: human milk oligosaccharide
IgE: immunoglobin E
LMM: linear mixed model
QC: quality control
RM‐ASCA+: repeated measures analysis of variance simultaneous component analysis+
SCORAD
: SCORing Atopic Dermatitis
Treg: regulatory T cell

## 1 Introduction

Cow's milk allergy (CMA), characterized by an immune‐mediated response to cow's milk (CM) protein(s), is one of the major food allergies in early life [1, 2]. Over the past decades, the estimated CMA prevalence in children of developed countries is approximately 0.5%–3% [3, 4]. The allergic symptoms typically occur in the first year of life, whereas the resolution age varies and is related to the type of CMA [5]. Based on symptoms and pathophysiology, CMA is categorized into immunoglobin E (IgE)‐mediated, non‐IgE mediated, and mixed IgE CMA [6]. Subjects with IgE‐mediated CMA, constituting approximately 60% of all CMA cases [3], require longer time for tolerance acquisition to CM than non‐IgE mediated CMA subjects [7, 8]. In recent decades, the relevance of the gut microbiome (GM) in CMA has been highlighted, and studies show that compared to healthy counterparts, children with IgE‐mediated CMA exhibit a reduction in bifidobacteria [9].

Bifidobacteria, the prototypical health‐promoting bacteria, are dominant inhabitants in a breastfed infant's gut [10] and play a pivotal role in GM development in early life [11, 12]. As coevolved bacteria, bifidobacteria possess unique glycosidases to digest complex host‐derived glycans, particularly the human milk oligosaccharides (HMOs) [13, 14]. The oligosaccharide fermentation products not only satisfy the energy and carbon demands of bifidobacteria but also benefit other bacteria through cross‐feeding activities, thereby contributing to maintaining the GM homeostasis in early life [10, 11].

Thus, bifidobacteria‐related probiotics and HMO‐mimicked prebiotics have gained popularity in the management of CMA in early life, alongside the conventional interventions with extensively hydrolyzed formula or amino acid‐based formula (AAF) [15]. Indigestible oligosaccharides, such as fructooligosaccharides (FOS) and galactooligosaccharides, are used as prebiotics due to their bifidogenic effect on the GM [16]. *Bifidobacterium* species, including *B. bifidum* [17], *B. longum* [18], and particularly *B. breve* [18, 19, 20, 21], are widely used probiotics for IgE‐mediated CMA management in infants. These bifidobacteria have key immunomodulatory roles in the cross‐talk between GM and host immune system: *B. bifidum*, for example, can induce the expression of FoxP3 in the regulatory T (T_reg_) cells through cell surface polysaccharides [22], while *B. longum* in neonatal microbiota can alleviate the risk of allergy by promoting the T_reg_ maturation [23]; *B. breve*, particularly the *B. breve* M‐16 V, can trigger the antiallergic process in early infancy by regulating the intestinal microbiota, intestinal epithelial barrier, and immune system [24]. Overall, bifidobacteria with HMO‐utilization genes are found to induce intestinal IFN‐β and silence Th2 and Th17 cytokines, thereby regulating the systemic immune balance in infants [25]. Additionally, by breaking down HMOs, bifidobacteria can indirectly enhance the production of butyrate [26] which is essential for the interplay between GM and systemic immunity [27], possibly through epigenetics mechanisms [28]. Bifidobacteria‐derived indolelactic acid (ILA) also actively engages in the immunoregulation during infancy [25, 29]. However, despite these findings and the wide application of bifidobacteria‐related interventions for IgE‐mediated CMA [17, 18, 19, 20, 21], none of the studies have reported comprehensive metabolome exploration.

In this study, we investigated longitudinal fecal metabolome changes of infants with IgE‐mediated CMA undergoing dietary management with AAF, with and without synbiotic (*Bifidobacterium breve* M‐16 V; FOS: oligofructose, inulin). By applying linear mixed models (LMMs) and repeated measures analysis of variance simultaneous component analysis+ (RM‐ASCA+), we compared the longitudinal fecal metabolome of infants with persistent CMA to those who developed CM‐tolerance and identified key metabolic changes associated with the synbiotic intervention.

## 2 Experimental Section

### 2.1 Study Design and Dosage Information

This study arises from a multicenter, randomized, double‐blind, controlled clinical study PRESTO (registered as NTR3725 in Netherlands Trial Register). Detailed information on ethics committees, institutional review boards, and regulatory authorities that approved the study was previously published [30].

PRESTO enrolled infants diagnosed with IgE‐mediated CMA who then received either AAF (Nutricia, Liverpool, UK) or amino acid‐based formula with synbiotic (AAF‐S) to manage their CMA. The synbiotic blend consisted of chicory‐derived neutral FOS: oligofructose and inulin in a 9:1 ratio (total concentration of 0.63 g/100 mL formula, BENEO‐Orafti SA, Oreye, Belgium) and *B. breve* M‐16 V (1.47 × 10^9^ cfu/100 mL formula, Morinaga Milk Industry, Tokyo, Japan). Caretakers were instructed to provide subjects with a minimum daily dose of 450, 350, and 250 mL for infants aged 0–8, 9–18, and older than 18 months, respectively [19]. After 12 months of intervention, the allergy status was reevaluated through a double‐blind, placebo‐controlled food challenge (DBPCFC) with CM. Detailed information on the diagnosis and reassessment was previously published [19]. Out of the 169 participants enrolled in PRESTO, 40 subjects (aged 3–13 months) were selected for this study based on sample availability. One subject was excluded due to unclear allergy status after 12 months [30]. Of the 16 AAF and 23 AAF‐S participants, 10 and 14 infants, respectively, outgrew CMA within 12 months. Stool samples were available at 0 (baseline, TP0), 6 (TP1), and 12 months (TP2) after the start of the intervention, resulting in a total of 117 samples.

### 2.2 Sample Collection and Storage

The sample collection procedure has been described previously [30]. In short, fecal samples were collected at home and immediately stored in freezers, then transferred on ice to the participant hospitals and stored at −80°C until transfer to Danone Research & Innovation (Utrecht, The Netherlands) for wet sample aliquoting and SCFAs and lactic acid analysis. Sample aliquots for LC‐MS metabolomics analysis were transferred on dry ice to Leiden University and stored at −80°C until analysis.

### 2.3 Metabolomic Analysis

#### 2.3.1 SCFAs and Lactic Acid Analysis

Quantitative SCFAs, including branched short‐chain fatty acid (BSCFA) analysis, was performed using GC coupled to flame ionization detector, and lactic acid was measured using a lactic acid assay kit (Megazyme, Wicklow, Ireland) as previously described [31].

#### 2.3.2 LC‐MS Metabolomics Analysis

The wet sample aliquots were lyophilized at 4 mbar and −110°C for 20 h (Martin Christ Gefriertrocknungsanlagen GmbH, Germany), weighed (20 ± 0.2 mg), and stored at −80°C until extraction. Liquid–liquid extraction was performed as described by Hosseinkhani et al. [32] with an adjusted sample amount and doubled solvent‐to‐feces ratio. Detailed information on the chemicals, the sample preparation, and the quality control (QC) is available in the Supporting Information.

Polar to semi‐polar metabolites, including acetylcarnitines, amines, benzenoids, organic acids, indoles, nucleosides, and nucleotides, were analyzed using reverse‐phase LC coupled with quadrupole (Q)‐TOF‐MS operated in full‐scan positive and negative ionization modes, as described previously [33] and in the Supporting Information. Bile and fatty acids were measured using reverse‐phase LC separation and Q‐TOF‐MS operated in full scan negative ionization mode, as described in the Supporting Information.

Targeted peak integration was performed using SCIEX OS (version 2.1.6., SCIEX) with a maximum mass error of 10 ppm. The retention times were verified against authentic standards. In case of coelution, the targets were reported using the name or abbreviation of one of the targets followed by a “#”. Details on the abbreviations used are listed in Table S2. For the polar to semipolar metabolites, peak area was used for further data analysis, whereas for the bile and fatty acids, the area ratio of compounds to stable isotopically labeled standards (Table S1) was used. Data quality inspection was performed using an in‐house quality assurance software performing between batch correction and removal of metabolites with high technical variance (RSD of QC > 30%).

#### 2.3.3 Data Analysis

Data handling and statistical analyses were performed in R (version 4.3.2). Metabolites with missingness above 20% and with the median signal of the samples less than five times the mean signal of the procedure blanks were removed, leaving 166 metabolites. To identify group bias in missingness, Fisher's exact test was performed for metabolites with missingness above 20% at each time point after grouping the subjects by intervention or CM‐tolerance status, and the results are summarized in Table S2. Ratios of secondary to primary and unconjugated to conjugated bile acids (BAs) were added, resulting in a total of 177 variables. A list of the reported metabolites and their abbreviations can be found in Table S3. The raw data were normalized by dry weight and subsequently log_2_‐transformed. Missing values were imputed per metabolite using the quantile regression imputation of left‐censored (QRILC) method [34]. Available clinical characteristics that potentially associated with CM‐tolerance status at TP2 or intervention were analyzed with the two‐sided Mann–Whitney *U* test for numeric variables and the Fisher's exact test for binary variables as reported previously [30, 35].

To assess the change from TP0 to TP1 and TP2, LMMs were built using the lme4 package in R. Before building the model, the data was scaled by the standard deviation of all baseline samples. The metabolites were modeled as response variables with group and time as fixed effects and subject ID as a random effect. After grouping the subjects by either their CM‐tolerance status at TP2 (CM‐allergic vs. CM‐tolerant) or intervention (AAF vs. AAF‐S), two models were built, namely tolerance‐allergy and intervention. For the tolerance‐allergy model (*Metabolite* *∼ time + CM‐tolerance_status + time:CM‐tolerance_status + (1|ID))*, TP0 and the CM‐allergic group were used as references. Pairwise comparisons between groups at each time point and within a group between the time points were performed using the emmeans package in R. For the intervention model (*Metabolite ∼ time + time:intervention + (1|ID)*), TP0 and the AAF group were used as references. The main effect of the intervention was removed from the model but its interaction with time was kept ensuring the groups are equal at baseline. The *p* values were calculated to assess a change from baseline with the Satterthwaite's degrees of freedom method using the lmerTest package within the ALASCA package [36]. In this study, the combined CM‐tolerance status–intervention model was not performed because CM‐tolerance acquisition as investigated in the parent study did not differ between the interventions at TP2 and aligned with natural rates of CMA outgrowth in infants [19]. For most metabolites, the addition of age as a covariate to models led to no improvement of the performance based on Akaike information criterion (Tables S4 and S5). Therefore, age was not used as a covariate in the LMMs. Multiple testing correction was performed using the Benjamini–Hochberg method where Q < 0.1 was considered as statistically significant.

Visualization of the longitudinal metabolomic alterations was achieved using RM‐ASCA+ with ALASCA package [36], as detailed in the Supporting information. Performances of the analysis were validated using nonparametric bootstrapping, and the 95% confidence intervals (CIs) were estimated based on 1000 resampling iterations.

### 2.4 16S rRNA Gene Sequencing and Preprocessing

Extraction of DNA from stool samples and the subsequent gut microbiota profiling by 16S rRNA gene sequencing were performed as described previously [30]. Correlations between the changes in metabolites and the relative abundance of *Bifidobacterium* were examined using Spearman's rank correlation analysis. Relative abundance comparisons of *Bifidobacterium* between and within the AAF and AAF‐S groups were evaluated with two‐sided unpaired *t* tests.

## 3 Results

### 3.1 Patient Characteristics

The statistical results of important clinical characteristics are summarized in Tables S6, S7. When grouping the subjects by the CM‐tolerance status at TP2, the father allergy occurrence and the SCORing Atopic Dermatitis (SCORAD) at baseline were significantly higher in the CM‐allergic group than in the CM‐tolerant group (Table S6). None of the clinical characteristics were significantly different between AAF and AAF‐S groups (Table S7).

### 3.2 More Pronounced Fecal Metabolome Changes in the CM‐Tolerant Group

**FIGURE 1:**
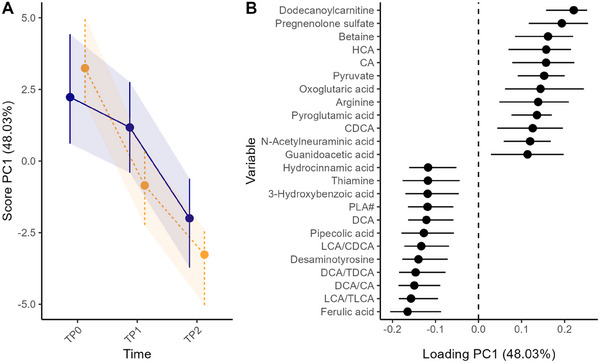
RM‐ASCA+ combined effect matrix showing the common metabolome development throughout the study for the CM‐allergic (blue solid line, *n* = 15) and CM‐tolerant (orange dashed line, *n* = 24) groups as scores (A) and loadings (B). Only the metabolites with 12 highest and 12 lowest loadings are shown in the plot. Error bars representing 95% CI were estimated based nonparametric bootstrapping. CI, confidence interval; CM, cow's milk; RM‐ASCA+, repeated measures analysis of variance simultaneous component analysis+.

Firstly, RM‐ASCA+ was used to examine the longitudinal metabolome alterations within and between infants that remained allergic and those that acquired tolerance to CM by TP2 (CM‐allergic vs. CM‐tolerant). The PC1 score plot (Figure 1A) describes the direction of maximum variance in the modeled data, whereas the loadings plot (Figure 1B) highlights the top metabolites contributing to PC1. Metabolites with positive loadings follow the trend described by the score, whereas the opposite holds for metabolites with negative loadings. Figure 1B shows that almost half of the variation (47%) described by the fixed effects of the tolerance‐allergy model was explained by PC1 (Figure 1A). The scores and loading for PC1 showe that over time ferulic acid, desaminotyrosine, pipecolic acid, and 3‐hydroxybenzoic acid increased, whereas dodecanoylcarnitine, pregnenolone sulfate, betaine, and pyruvate decreased (Figure 1). Few BAs also showed overall change with time. The primary BAs cholic acid (CA), chenodeoxycholic acid (CDCA), and hyocholic acid (HCA) declined over time. In contrast, the secondary BAs deoxycholic acid (DCA) and the ratios of secondary to primary BAs, including DCA/CA, lithocholic acid (LCA)/CDCA, increased. Although with overlapped CIs between the two groups, those changes were more pronounced for the CM‐tolerant group where the PC1 score declined more sharply than the CM‐allergy group and for which the CIs between the time points were separated, suggesting a significant time effect in this group.

Univariate marginal means comparison showed that around five times more metabolites were significantly altered over time in infants that acquired CM‐tolerance versus those that remained CM‐allergic (TP0–TP1: 9 metabolites in CM‐tolerant vs. 2 metabolites in CM‐allergic; TP0–TP2: 30 metabolites in CM‐tolerant and 7 in CM‐allergic; Figure S1 and Table S8). Pregnenolone sulfate, pyroglutamic acid, pyruvate, oxoglutaric acid, and ferulic acid were significantly affected by time for both groups and followed comparable time‐development trends (Figure S1). Similarly, arginine decreased, whereas 3‐hydroxybenzoic acid, hydrocinnamic acid, LCA, and DCA increased simultaneously in both groups, but significantly only in the CM‐tolerant group (Figure S1). Pipecolic acid levels increased over time in both groups, but the rise was steeper and significant only in the CM‐tolerant group. Dodecanoylcarnitine followed the trend described by PC1 of the combined effect matrix (Figure 1A) with a decline in time at both TP1 and TP2 significant only in the CM‐tolerant group. The rest of the significantly altered metabolites showed dissimilar longitudinal profiles between the groups (Figure S1). Butyric acid, PLA#, desaminotyrosine, and phenylacetic acid were significantly increased, whereas 5‐hydroxytryptophan and the primary BAs CA and CDCA showed significant decreases in the CM‐tolerant group only. In contrast, threonine#, and tryptophan significantly increased over time only in the CM‐allergic group.

**FIGURE 2:**
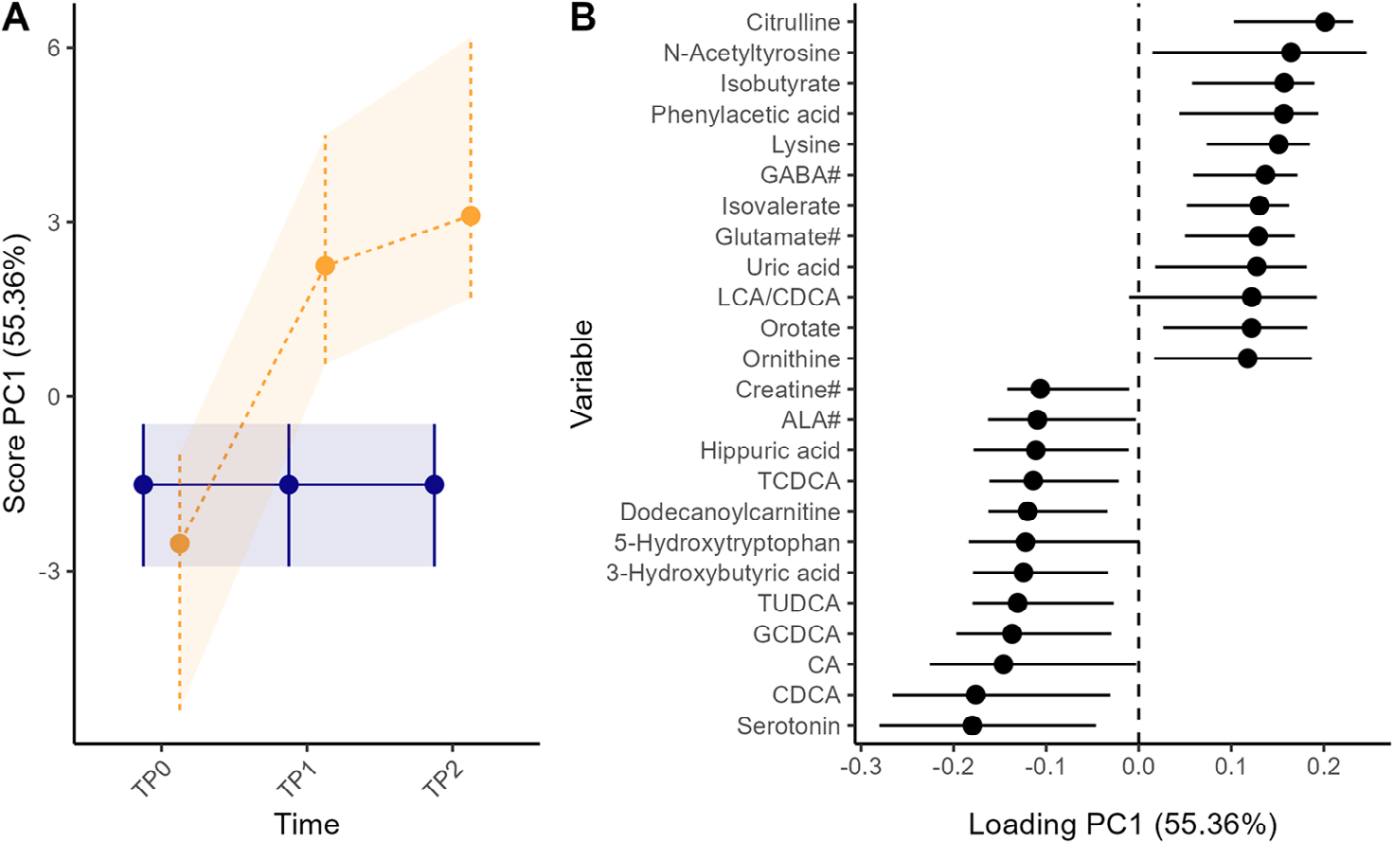
RM‐ASCA+ interaction effect matrix showing the metabolome differences between the CM‐allergic (blue solid line, *n* = 15) and CM‐tolerant group (orange dashed line, *n* = 24) over time as scores (A) and loadings (B). Only the metabolites with 12 highest and 12 lowest loadings are shown in the plot. Error bars representing 95% CI were estimated based nonparametric bootstrapping. CI, confidence interval; CM, cow's milk; RM‐ASCA+, repeated measures analysis of variance simultaneous component analysis+.

Next, the RM‐ASCA+ interaction effect matrix was examined to focus on the alterations associated with CM‐tolerance acquisition. The PC1 scores and loading of the interaction matrix, Figure 2, suggest that compared to the CM‐allergic group, the CM‐tolerant group showed overall alterations in amino acid metabolism with an increase in citrulline, lysine, *N*‐acetyltyrosine, phenylacetic acid, gamma‐aminobutyric acid (GABA#), glutamate, orotate, and ornithine as well as a decrease in 5‐hydroxytryptophan and serotonin. The BAs metabolism was also altered: decline in CDCA, CA, glycochenodeoxycholic acid (GCDCA), tauroursodeoxycholic acid (TUDCA), taurochenodeoxycholic acid (TCDCA) and increase in LCA/CDCA for the CM‐tolerant group. The BSCFAs, isobutyrate and isovalerate, also contributed to PC1, showing higher levels in the CM‐tolerant group. However, only citrulline and lysine were found significantly different at TP2 between the two groups univariately (Table S6, Figure S2).

### 3.3 Synbiotic Supplementation Altered Fecal Metabolome After Six Months of Intervention

**FIGURE 3:**
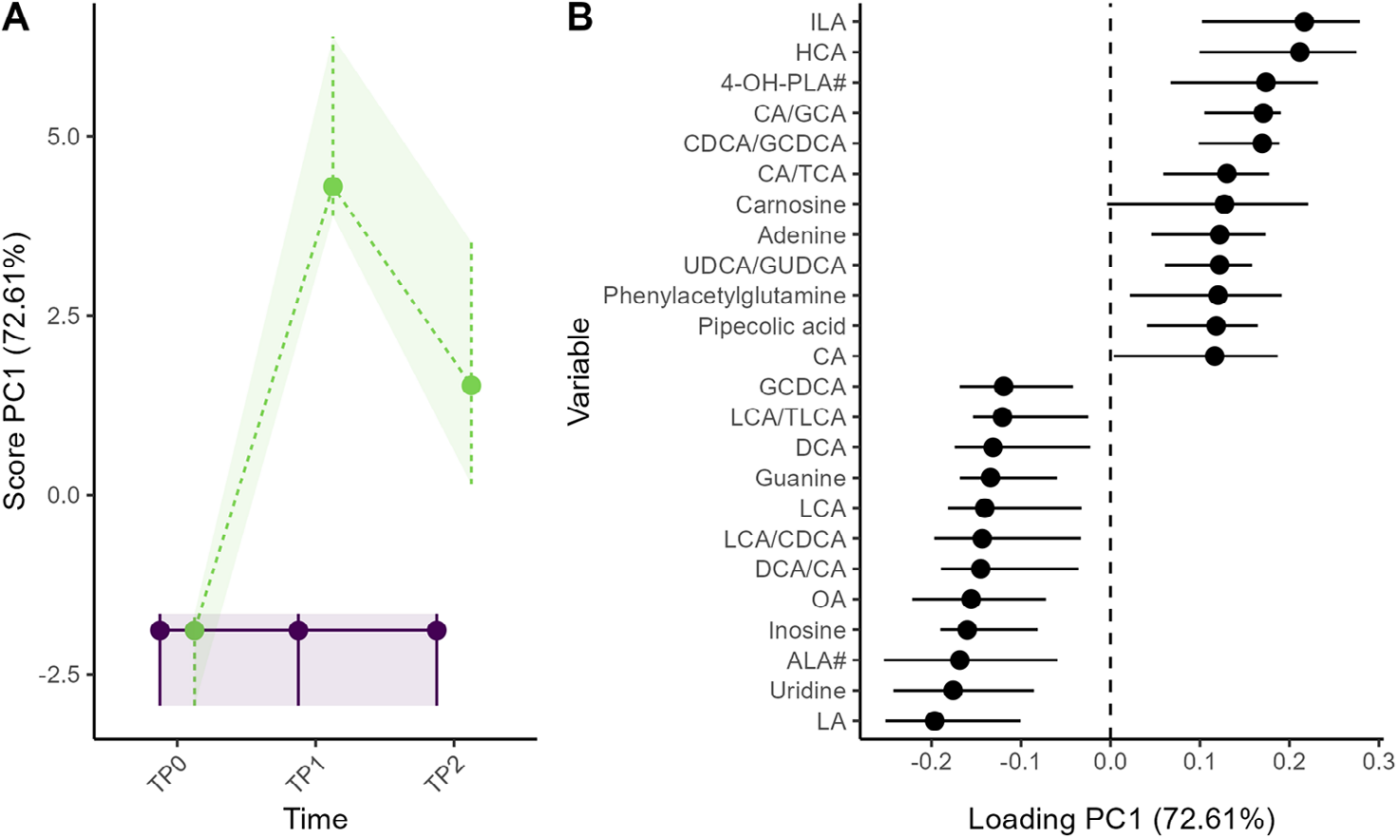
RM‐ASCA+ interaction effect matrix showing the metabolome differences between the AAF (purple solid line, *n* = 16) and AAF‐S (green dashed line, *n* = 23) group over time as scores (A) and loadings (B). Only the metabolites with 12 highest and 12 lowest loadings are shown in the plot. Error bars representing 95% CI were estimated based nonparametric bootstrapping. AAF, amino acid‐based formula; AAF‐S, amino acid‐based formula with synbiotic; CI, confidence interval; RM‐ASCA+, repeated measures analysis of variance simultaneous component analysis+.

The longitudinal alterations of the fecal metabolome between the AAF and AAF‐S group were studied to understand the effect of the synbiotic supplementation. As shown in Figure 3, clear group separation was observed in PC1 of the RM‐ASCA+ interaction effect matrix, especially at TP1.

**FIGURE 4:**
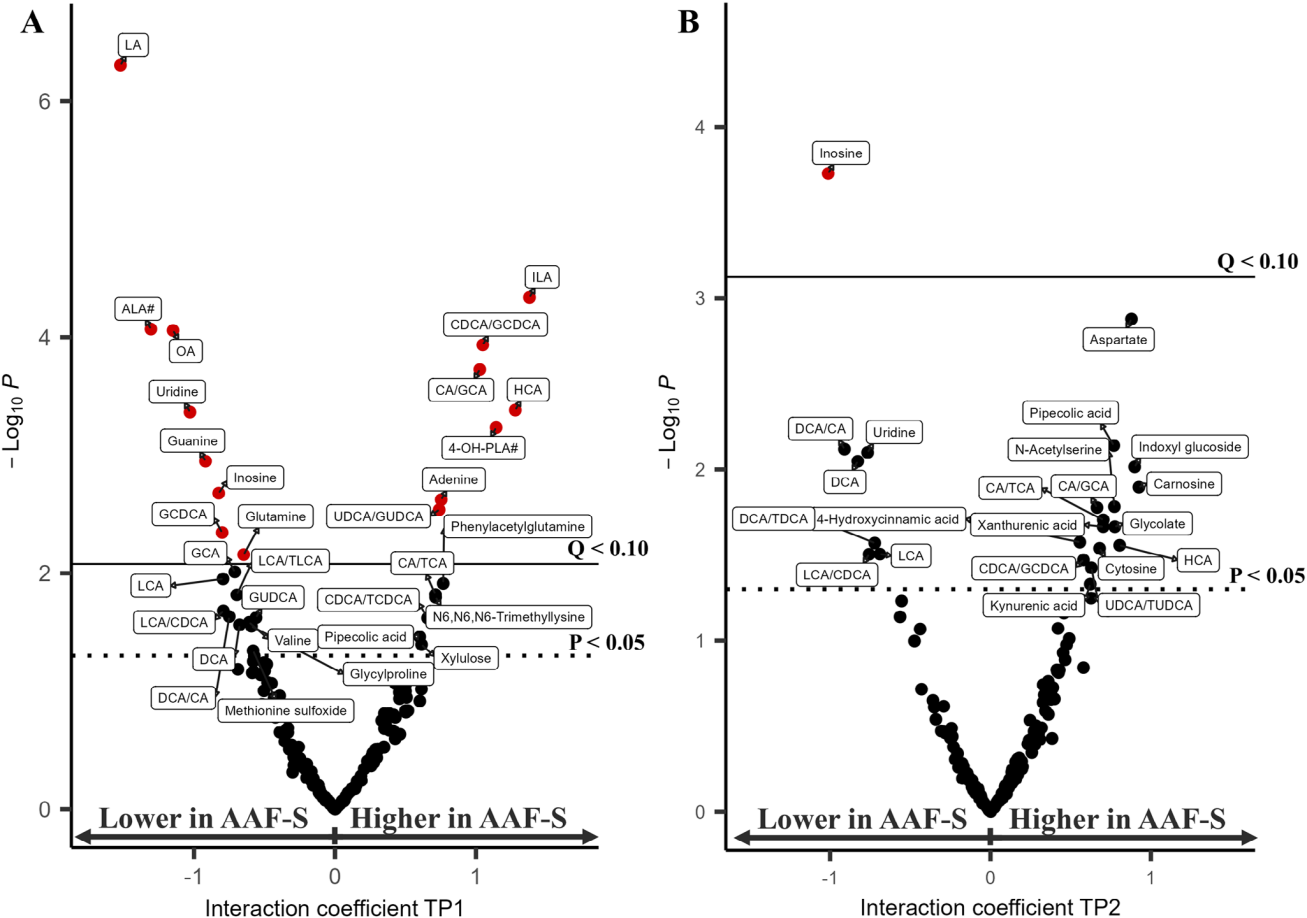
Volcano plot showing the resulting *p* value of the interaction coefficient for TP1 (left) and TP2 (right) in intervention LMM, dashed (*p* = 0.05), solid line (*Q* = 0.1) for TP1 (A) and TP2 (B). Red symbols indicate metabolites with *Q* < 0.1 after Benjamini–Hochberg procedure. LMM, linear mixed model.

Among all the metabolites, 12 metabolites and three BA ratios were found to be statistically different between the AAF and AAF‐S groups at TP1, and only inosine at TP2 (Figure S3, Table S8). The estimated marginal means plot of those analytes can be found in Figure S3. The synbiotic supplementation led to an increase of gut microbial metabolites ILA and 4‐hydoxyphenyllactic acid (4‐OH‐PLA#) and a decline in the fatty acids linoleic acid (LA), alpha‐linolenic acid (ALA#), and oleic acid (OA) at TP1 (Figure 4). Amino acid glutamine was also decreased in the AAF‐S group at TP1. Three purine metabolites inosine, guanine, and adenine as well as the pyrimidine uridine were also affected by the intervention. Although adenine was higher upon the synbiotic addition, the opposite was true for inosine, guanine, and uridine. HCA and CDCA/GCDCA, CA/glycocholic acid (GCA), and ursodeoxycholic acid (UDCA)/glycoursodeoxycholic acid (GUDCA) were all significantly higher in the AAF‐S than in the AAF group at TP1, whereas GCDCA was significantly lower (Figure 4). A few other BAs were found to be among the main contributors to PC1 of the interaction matrix (Figure 3) or to have significant interaction coefficient at TP1 before multiple testing corrections (Figure 4), namely, the glyco‐conjugated BAs GCA and GUDCA and the secondary BAs and their ratio to primary BAs: LCA, DCA, DCA/CA, and LCA/CDCA.

### 3.4 Association Between Changes in *Bifidobacterium* and Metabolites Significantly Altered by the Synbiotic

**FIGURE 5:**
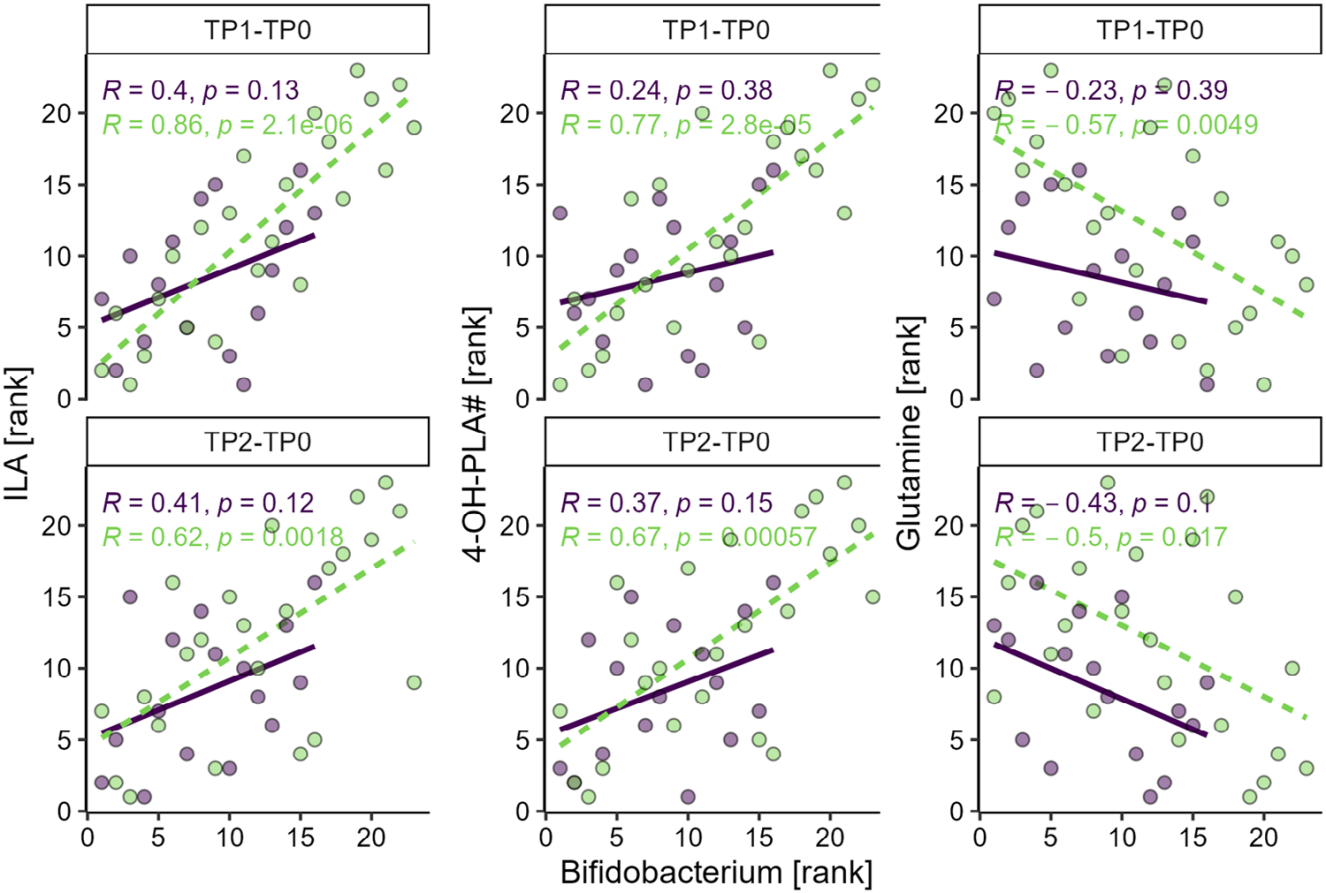
Spearman's rank correlations between the changes in *Bifidobacterium* and ILA, 4‐OH‐PLA#, glutamine in AAF (purple solid line, *n* = 16) and AAF‐S (green dashed line, *n* = 23) groups from baseline to TP1 (TP1–TP0) and TP2 (TP2–TP0). The rank of the changes in metabolite response and relative abundance of *Bifidobacterium* within each group were used for plotting. The figure shows *p* values; the *Q* values after Benjamini–Hochberg procedure are provided in Table S9. 4‐OH‐PLA#, 4‐hydoxyphenyllactic acid; AAF, amino acid‐based formula; AAF‐S, amino acid‐based formula with synbiotic; ILA, indolelactic acid.

The synbiotic supplementation significantly increased the relative abundance of *Bifidobacterium* in the AAF‐S group from baseline to TP1 and TP2 compared to the AAF group (Figure S4) [35]. To determine whether these increases were associated with the significantly changed metabolites, Spearman's rank correlation analysis was performed between the changes in metabolite levels and *Bifidobacterium*’s relative abundance from baseline to TP1 (TP1–TP0) and TP2 (TP2–TP0), respectively (Table S9). In the AAF‐S group, changes in ILA and 4‐OH‐PLA# from TP0 to later time points were positively correlated with those of *Bifidobacterium* (*r* > 0.6, *p* < 0.005), while changes in glutamine were negatively correlated (*r* ≤ −0.5, *p* < 0.05) (Figure 5). The changes in *Bifidobacterium* were positively correlated with those of adenine at TP1 and TP2 in both groups (*r* > 0.5, *p* < 0.05), and with CDCA/GCDCA and CA/GCA only at TP1 in the AAF‐S group (*r* > 0.4, *p* < 0.05). *Bifidobacterium* also showed negative correlations with GCDCA and inosine in changes from TP0 to TP1 only in the AAF‐S group (*r* < −0.4, *p* < 0.05) (Figure S5).

## 4 Discussion

In this study, we followed the fecal metabolome alterations in infants with IgE‐mediated CMA who received AAF with or without synbiotics for a year. Firstly, we examined the effect of CM‐tolerance acquisition on the fecal metabolome over time. Time, reflecting growth and diet diversification, had a more pronounced impact on the metabolome than CM‐tolerance acquisition (Figure 1, Figure S1). The diet enrichment was evidenced by the overall increase of the phenolic acids, which are ubiquitously produced in plants [37], including ferulic acid, 3‐hydroxybenzoic acid, and hydrocinnamic acid. The decrease in the steroid hormone (pregnenolone sulfate), energy metabolites (pyruvate, oxoglutaric acid, and dodecanoylcarnitine), and the altered amino acids and derivatives (pyroglutamic acid, arginine, and pipecolic acid) suggest metabolome modification associated with somatic growth [38, 39].

The multivariate RM‐ASCA+ analysis showed an association of CM‐tolerance acquisition status with alterations in amino acids, BAs, and (B)SCFAs (Figure 2). Compared to infants with persistent CMA, citrulline and lysine were significantly higher in the infants who developed CM‐tolerance at TP2 (Figure S2). Lower plasma citrulline levels are known markers of increased gut permeability [40], which can raise the chance of allergen(s) passing the intestinal barrier and triggering the immune system [41]. The increase in fecal citrulline in the CM‐tolerant group in this study might suggest improved gut barrier function and gut health. Although not significantly different between the two groups, the amino acids GABA#, glutamate#, threonine#, and ornithine were also higher in the CM‐tolerant group compared to the CM‐allergic group (Figures S1–S2). Lower fecal threonine levels have previously been reported in infants with IgE‐mediated CMA compared to healthy controls [42]. Interestingly, although not significant, 5‐hydroxytryptophan and serotonin were higher in the CM‐allergic group at TP1 and TP2 (Figure 2), while their precursor tryptophan significantly declined only from TP0 to TP2 in this group (Figure S1). As serotonin is involved in intestinal epithelial proliferation [43] and plays an essential role in regulating intestinal inflammation [44], the upregulated tryptophan–serotonin metabolism in the CM‐allergic group may reflect an inflammatory state of the intestine in the CMA infants.

Children who outgrew CMA showed differences in their BAs profile. The primary BAs (CA, CDCA) significantly decreased, while the secondary BAs (DCA, LCA) and the secondary/primary BAs ratios (DCA/CA, LCA/CDCA) significantly increased from TP0 to TP2 only in the CM‐tolerant group (Figure S1). A recent study found that, compared to healthy children, children with IgE‐mediated CMA had lower ratios of fecal secondary/primary BAs from the CA pathway, with DCA and other oxidized keto BAs included in the calculation [45]. Secondary BAs from the CDCA pathway, including LCA, were reported lower in children with food allergy compared to healthy controls as well [46]. Although the secondary BAs and secondary/primary BAs ratios were not significantly different between the two groups in our study, the altered BAs profiles in the CMA‐tolerant group likely indicate a more mature GM for secondary BAs production. This may contribute to improved intestinal functions in infants outgrowing CMA, as LCA is known to attenuate disruption in the intestinal barrier [47].

(B)SCFAs were also altered during the CMA tolerance acquisition process. Butyrate significantly increased from TP0 to TP2 only in the CM‐tolerant group (Figure S1). Isobutyrate and isovalerate tended to have group separation at TP1, with a continuous elevation in the CM‐tolerant group over time, and a decrease at TP1 in the CM‐allergic group (Figure S2). Consistent with our finding, those (B)SCFAs, specifically butyrate, are known for their antiinflammatory effects [27, 48] and are generally observed to be lower in the feces of children with IgE‐mediated food allergy [42, 48]. Additionally, phenylalanine, phenyllactic acid (PLA#), and desaminotyrosine, which are GM metabolites from amino acids and dietary polyphenols [49, 50, 51], were significantly increased from TP0 and TP2 only in the CM‐tolerant group (Figure S1). The significant elevations of these metabolites may promote CM‐tolerance acquisition, especially considering the recently recognized antiinflammatory property of desaminotyrosine [52, 53].

The synbiotic (*B. breve* M‐16 V, FOS: inulin, oligofructose) significantly altered the levels of aromatic lactic acids, purine metabolites as well as fatty acids and BAs, particularly after 6 months of intervention. The intervention enhanced ILA and 4‐OH‐PLA levels (Figure S3), and their increases from baseline to TP1 and TP2 were positively correlated with those of bifidobacteria (Figure 5). This finding aligns with reports that ILA and 4‐OH‐PLA are metabolites of tryptophan [29, 54, 55] and tyrosine [29] produced by infant‐type *Bifidobacterium* species, including *B. breve*. Earlier published microbiome and metaproteomics analysis of stool samples from the same clinical trial revealed that the synbiotic raised the level of bifidobacteria [19, 35], as well as bifidobacterial Carbohydrate‐Active enZymes [35], known to metabolize FOS [56]. Although the proportion of *Bifidobacterium* was significantly higher in the AAF‐S group compared to the AAF group at both time points (Figure S4) [19, 35], the increases in ILA and 4‐OH‐PLA# were significantly higher in the AAF‐S group only at TP1. These results suggest that the synbiotic promoted the growth and/or the activity of aromatic lactic acid producers, for example, infant‐type *Bifidobacterium* species, especially at TP1. This can be evidenced by stronger positive correlations between changes in the two aromatic lactic acids and bifidobacteria from baseline to TP1 than to TP2 in the AAF‐S group (Figure 5). To validate our observations, *Bifidobacterium* species should be quantified. Alternatively, aromatic lactate dehydrogenase reported to convert tryptophan and tyrosine to respectively ILA and 4‐OH‐PLA in infant‐type *Bifidobacterium* species should be analyzed [29]. The possibility that the ILA and 4‐OH‐PLA# were produced by some lactic acid bacteria should not be ignored either [57, 58]. Overall, the increased ILA and 4‐OH‐PLA# levels in the AAF‐S group suggest enhanced abundance or activity of infant‐type bifidobacteria, supporting the successful synbiotic supplementation together with the microbiome and metaproteomics findings [19, 35]. Although the parent study found that the CM‐tolerance acquisition after 12 (TP2) and 24 months of synbiotic intervention aligned with natural outgrowth [19], our findings, along with the reported antiinflammatory effect of ILA [25, 29, 55, 59] suggest that the synbiotic intervention may pose beneficial effects on infants’ immune system. Further metabolomics studies on larger cohorts are required to verify this hypothesis.

In addition to the increase in ILA and 4‐OH‐PLA, the synbiotic lowered inosine, guanine, and uridine and raised adenine levels. The same purine‐pyrimidine trend was observed in conventionally raised and core microbiota‐colonized mice in comparison to germ‐free mice, indicating the importance of the GM in purine and pyrimidine metabolism [60]. A decline of inosine and uridine has also been reported in coculture of *B. breve* with small intestinal‐like epithelial cells [61]. *Lactobacillus brevis*, belonging to the *Lactobacillaceae* family, was found to be elevated in the AAF‐S group for the same set of samples [35] and was also reported to have inosine degradation capabilities [62]. To link the purine–pyrimidine metabolism to the GM, and the role of *Bifidobacterium* spp. and *Lactobacillaceae* spp. herein, more research is required.

The AAF‐S intervention lowered LA, ALA#, and OA levels, suggesting high consumption of these fatty acids by gut bacteria. This may be a result of hydration by bacteria of the *Lactobacillus* and *Bifidobacterium* genera [63] or the production of conjugated fatty acids [64, 65, 66, 67, 68]. *Bifidobacterium* strains, especially *B. breve*, are among the best producers of conjugated LAs [66, 67] and conjugated linolenic acids [66, 68].

The synbiotic enhanced the deconjugation of BAs, especially at TP1, where significantly decreased GCDCA and increased CDCA/GCDCA, CA/GCA, and UDCA/GUDCA were observed in the AAF‐S compared to the AAF group (Figure 4). *Bifidobacterium*, in general, are active bile salt hydrolase (BSH) producers [69], which perform preferred deconjugation activity on glyco‐conjugated BAs [70]. This aligns with our results showing that *Bifidobacterium* changes from baseline correlated negatively with those of GCDCA, and positively with those of CA/GCA and CDCA/GCDCA at TP1 in the AAF‐S (Figure S5). These correlations in changes disappeared at TP2, possibly due to increased GM diversity. Compared to TP0, families from other phyla, including Bacteroidetes, Firmicutes, and Proteobacteria, were more abundant at later timepoints in both groups, especially at TP2 [35]. These bacteria have also been identified as active BSH producers [71], thus might eliminate the correlation between the activity of BAs deconjugation and *Bifidobacterium*. Unexpectedly, the increased deconjugation activity of BAs failed to promote the production DCA and LCA. In contrast, although not significant, their levels and ratios to precursors (DCA/CA, LCA/CDCA) were lower in the AAF‐S than the AAF group (Figure 4). Considering that the conversion of primary BAs to secondary ones is highly conserved in bacteria with the *bai* operon [72] and that the host liver can further hydroxylate secondary BAs to tertiary BAs after gut–liver circulation [73], it is likely that more complex mechanisms underlie the host–gut metabolism of BAs during the intervention.

Our study has several limitations, including the wide age range of the participants at baseline of 3–13 (9.00 ± 2.90) months. Considering the rapid development of the GM in the first 2 years of life [39], the wide age range may obscure the observation of fecal metabolome alterations related to CM‐tolerance acquisition and the effect of intervention. Another limitation is the lack of information on the CM‐tolerance status at TP1. Knowing the status at TP1 could have aided in the interpretation of CM‐tolerance acquisition results. The research carried out for this paper is exploratory due to the small samples size (39 subjects). Increasing the sample size is necessary to verify these findings and would also allow to build LMM and RM‐ASCA+ models following the intervention and CM‐tolerance acquisition simultaneously. In addition, the parent study concluded that the synbiotic supplementation did not significantly affect CMA resolution. Thus, in this study, we cannot draw any conclusions regarding the clinical benefits of the synbiotic supplementation on CM‐tolerance acquisition based on fecal metabolome alterations. Despite those limitations, our study revealed several fecal metabolome pathway alterations that may contribute to CMA outgrowth. Most importantly, we found that the AAF‐S significantly altered the fecal metabolome after 6 months of the intervention, not after 12 months, suggesting that early intervention is required to maximize the effect of synbiotics. These findings aid in understanding the link between IgE‐mediated CMA‐tolerance acquisition, GM, and synbiotics intervention.

## Conflicts of Interest

Harm Wopereis is an employee of Danone Research & Innovation. The project is part of a partnership program between NWO‐TTW and Danone Research & Innovation. The other authors declare no conflicts of interest.

## Supporting information



Supporting Information

Supporting Information

## Data Availability

The data that support the findings of this study are openly available in MetaboLights at http://www.ebi.ac.uk/metabolights/, reference number.
