# Supplementary material for: Exploring the Fecal Metabolome in Infants With Cow's Milk Allergy: The Distinct Impacts of Cow's Milk Protein Tolerance Acquisition and of Synbiotic Supplementation
Source: Mol Nutr Food Res. 2024 Dec 12;69(1):e202400583. doi: 10.1002/mnfr.202400583 (PMC11704826; doi:10.1002/mnfr.202400583)
Supplement: Supplementary file 1 — Supporting Information [file MNFR-69-e202400583-s001.docx]

**Supplementary information**

**The impact of synbiotic supplementation and cow’s milk protein tolerance acquisition on the fecal metabolome in infants with cow’s milk allergy**

Authors: Pingping Zhu^1,#^, Mariyana V. Savova^1,#^, Alida Kindt^1^, the PRESTO study team, Harm Wopereis^2^, Clara Belzer^3^, Amy C. Harms^1*^, Thomas Hankemeier^1^

^1^Metabolomics and Analytics Centre, Leiden Academic Centre for Drug Research, Leiden University, Leiden 2333 CC, Netherlands

^2^Danone Research & Innovation, Uppsalalaan 12, 3584 CT Utrecht, the Netherlands

^3^Laboratory of Microbiology, Wageningen University, Stippeneng 4, 6708 WE Wageningen, the Netherlands

#Shared first authorship

^*^Corresponding author: Dr. Amy C. Harms, Metabolomics and Analytics Centre, Leiden Academic Centre for Drug Research, Leiden University, Leiden 2333 CC, Netherlands

E-mail: [a.c.harms@lacdr.leidenuniv.nl](mailto:a.c.harms@lacdr.leidenuniv.nl) , Tel: +31 623601213

**Supplementary methods**

**Chemicals**

Methyl tert-butyl ether (MTBE, ≥99.8%) and ammonium formate (≥99.0%) were purchased from Sigma Aldrich (St. Louis, United States). LC-MS-grade methanol (MeOH), isopropanol and formic acid (FA) were purchased from Biosolve B.V. (Valkenswaard, Netherlands). LC-MS grade acetonitrile was purchased from Actu-all chemicals (Randmeer, The Netherlands) and Biosolve B.V. (Valkenswaard, Netherlacnjugnds). Purified water was obtained from a Milli-Q PF Plus system (Merck Millipore, Burlington, United States). List of the isotopically labelled standards (SILs), including supplier details, can be found in Table S1.

**Sample preparation**

Briefly, 72 µL of water and 216 µL MeOH, containing stable isotopically labelled standards (SILs) (Table S1), were added to the 20 mg dry-weight fecal sample. After a 3-minute vortex mixing (Marshall Scientific, Cambridge, UK) 120 µL ice-cold MTBE was added, followed by another 3-minute vortex mixing. Following a brief centrifugation (30s, 100g, 4 °C), 200 µL of water and 168 µL of MTBE were added. The samples were vortex mixed for another 3 min, incubated at 4°C for 10 minutes until centrifugation (20 min, 16 000g, 4°C) inducing aqueous and organic layer separation. All solvents used during the LLE were ice-cold and vortex mixing was always at maximum speed. Following layer separation, each layer was transferred to an Eppendorf tube, followed by 5 and 2.5 minutes of centrifugation (16000g, 4°C) for aqueous and organic layers respectively. After extraction, 150 µL of the aqueous layer was aliquoted for polar to semi-polar metabolites analysis, while 48.8 μL of aqueous and 28.8 μL of organic layer was combined for the bile and fatty acids analysis. The aliquots were dried in a Speedvac (Labcono, USA) and stored at -80°C. Prior to LC-MS analysis, the extracts were reconstituted in 50 µL of 0.1% FA in water for polar to semi-polar metabolites analysis, and 200 µL of MeOH for the bile and fatty acids analysis. The reconstitution solvents contained different SILs (Table S1).

**Quality Control**

Samples were randomized into two batches, with those from the same subject prepared and measured in the same batch. For the preparation of the quality control sample, 30 study samples were weighed and extracted. After the extraction, equal volumes of each layer were taken from each sample and pooled, resulting in pooled QC aqueous and organic layers. Those pooled layers were used to prepare QC samples for each platform. The LLE and aliquoting steps were performed as described in Sample preparation.

**LC-MS analysis of polar to semi polar metabolites**

Analysis of polar to semi-polar metabolites were performed with a Shimadzu Nexera X2 LC system coupled to a TripleTOF 6600 mass spectrometer (SCIEX, Foster City, CA, USA), as described previously. Briefly, the LC separation was carried out at 40 °C using a Waters Acquity UPLC HSS T3 column (1.8 μm, 2.1 mm × 100 mm) with pre-column in-line stainless steel filter (0.3 μm, Agilent Technologies, Waldbronn, Germany). The mobile phase A was 0.1% FA in water, and the mobile phase B was 0.1% FA in ACN (Actu-all chemicals). With a flow rate of 0.4 mL min^-1^ and 1 μL of injection volume, the gradient starts at 100% A; 0–0.5 min 80% A; 0.5–2.5 min 2% A; 2.5–7.5 min 2% A; 7.5–12 min 2% A; 12 – 15 100% A. The data were acquired under full scan mode over the *m/z* range of 60-800 Da with Analyst TF software 1.7.1 (SCIEX) in negative and positive ionization modes. The preferred ionization mode for metabolites detectable in both polarities was chosen based on lower RSD% and higher signal-to-noise ratio of the QC samples.

**LC-MS analysis of bile acid and fatty acids**

Analysis of bile and fatty acids was performed on an UPLC-TOF/MS system consisting of ExionLC™ AC UHPLC system and SCIEX ZenoTOF 7600 system (Darmstadt, Germany) equipped with an IonDrive™ Turbo V Source, operated in negative ESI mode. The ion source conditions were as follows: spray voltage of 4.5 kV, capillary temperature of 550°C, ion source gas 1 50 psi, ion source gas 2 50 psi, curtain gas 35 psi, CAD gas 7 psi. The MS data was acquired under full scan mode over the m/z range of 200-900 Da. Accumulation time was set to 0.25 s, delustering potential to -70V and collision energy to -10eV. Chromatographic separation was performed on a Waters Acquity UPLC HSS T3 column (1.8 μm, 2.1 mm × 100 mm) with pre-column in-line stainless steel filter (0.3 μm, Agilent Technologies, Waldbronn, Germany). The flow rate was set at 0.4 ml min^-1^, the column was kept at 45 °C, injection volume at 2 μL. Mobile phase A consisted of 10 mM ammonium formate in water/ACN (Biosolve B.V) (95:5, v:v), while mobile phase B was 10 mM ammonium formate in MeOH/water (99:1, v:v). The gradient was as follows: starting at 0% B; 0–0.2 min 70% B; 0.2–7.5 min 100% B; 7.5–11.5 min 100% B; 11.5–11.6 min 0% B; 11.6 – 15 0% B. Isopropanol was used as an external rinsing solution (2 s sip time + rinse port). The flow was directed to waste in the first minute of the run. The autosampler temperature was set at 10 °C. Data acquisition was carried out on SCIEX OS 2.1.6.

**Visualization RM-ASCA+**

Visualization of the longitudinal metabolomic alterations was achieved using RM-ASCA+, which is an extension of LMMs for multivariate data. In the first step, LMMs are used to decompose the response matrix into effect matrices. The effect matrices are then analyzed using principal component analysis (PCA), and the results are summarized into PCA scores and loadings. The LMMs used for RM-ASCA+ were the LMMs used for the univariate analysis. The visualized effect matrices included the time effect matrix (‘time’) which shows time development of the reference group over time. The interaction matrix (‘time:group’) and the group-interaction matrix (‘group + time:group’) both show the deviations of the study group compared to the reference group over time with the latter also displaying the baseline differences. Lastly, the combined matrix (‘time + time:group’ or ‘time + group + time:group’) shows the time development of both the study and the reference group.


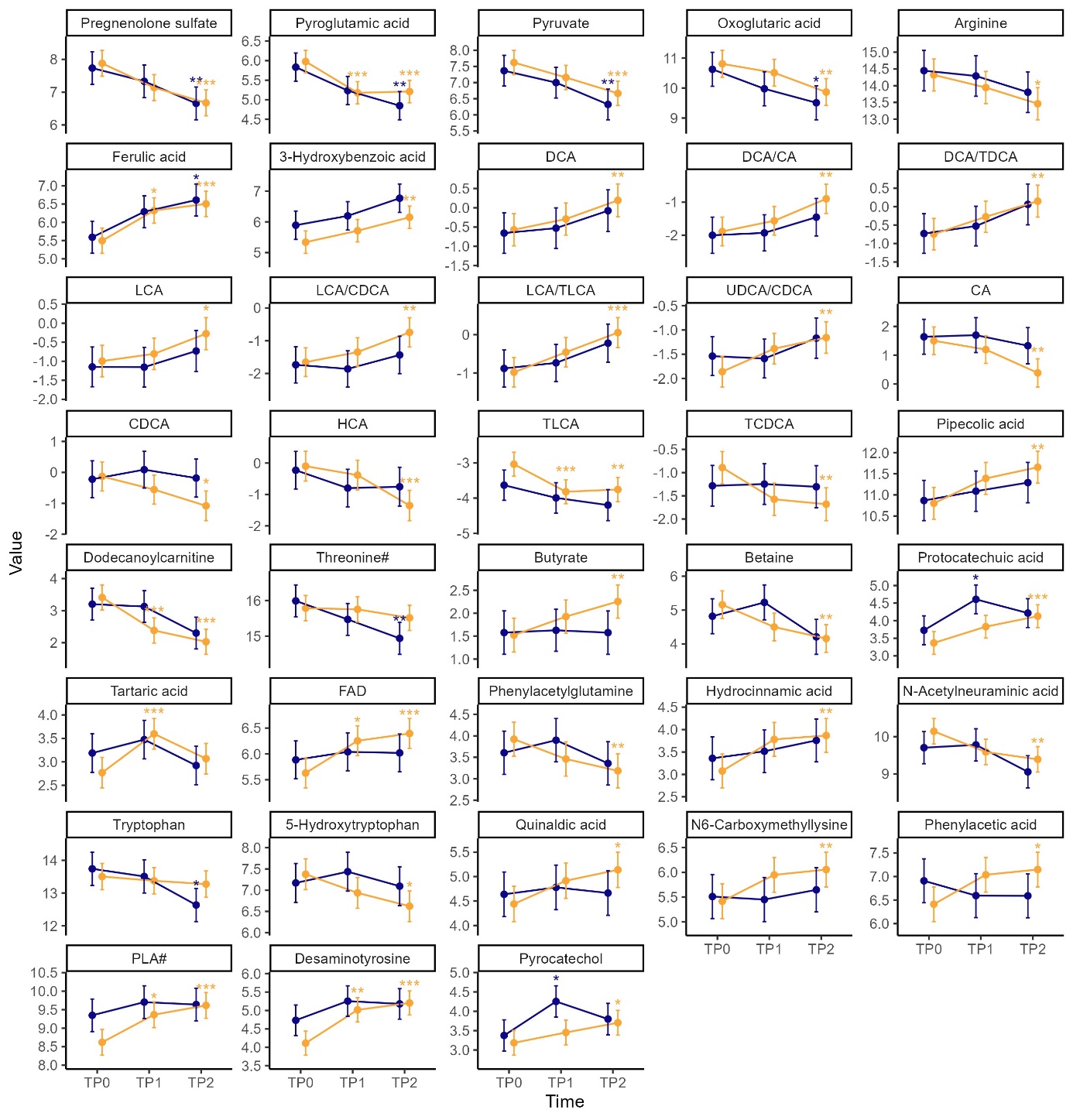


Figure S1. Marginal means estimated from the LMMs for participants who acquired tolerance (CM-tolerant, orange) and those that remained allergic (CM-allergic, blue). Only the metabolites for which pairwise comparison in time was found significant are plotted. The q-values are based on the marginal mean comparison to TP0 for each group, q < 0.01 (***), q < 0.05 (**), q <0.1 (*).


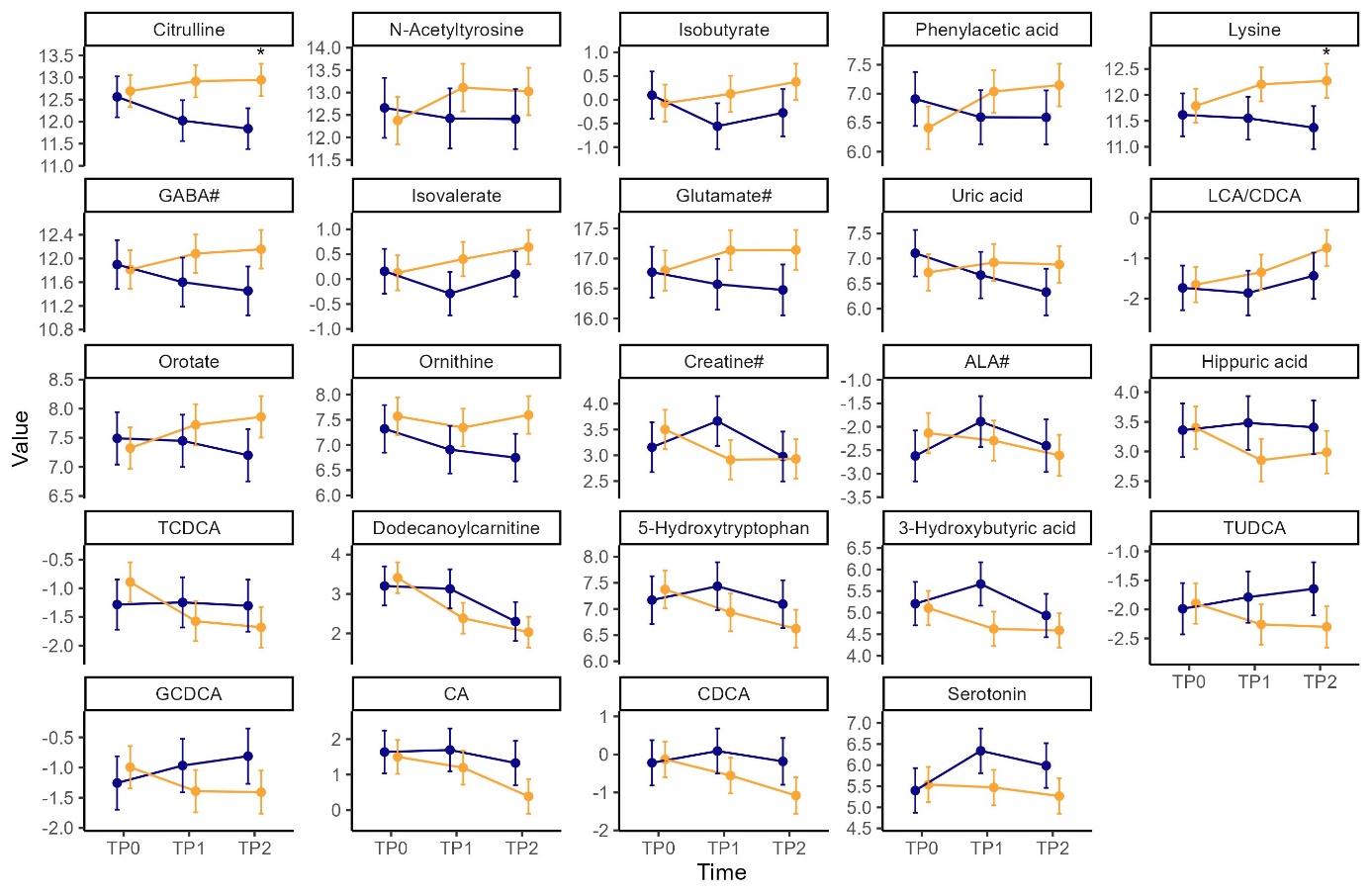


Figure S2. Marginal means estimated from the LMMs for participants who acquired tolerance (CM-tolerant) and those that remained allergic (CM-allergic). The metabolites with top loadings in PC1 of the RM-ASCA+ interaction matrix are plotted. The q-values are based on the marginal mean comparison between the groups at each time point, q <0.1 (*).


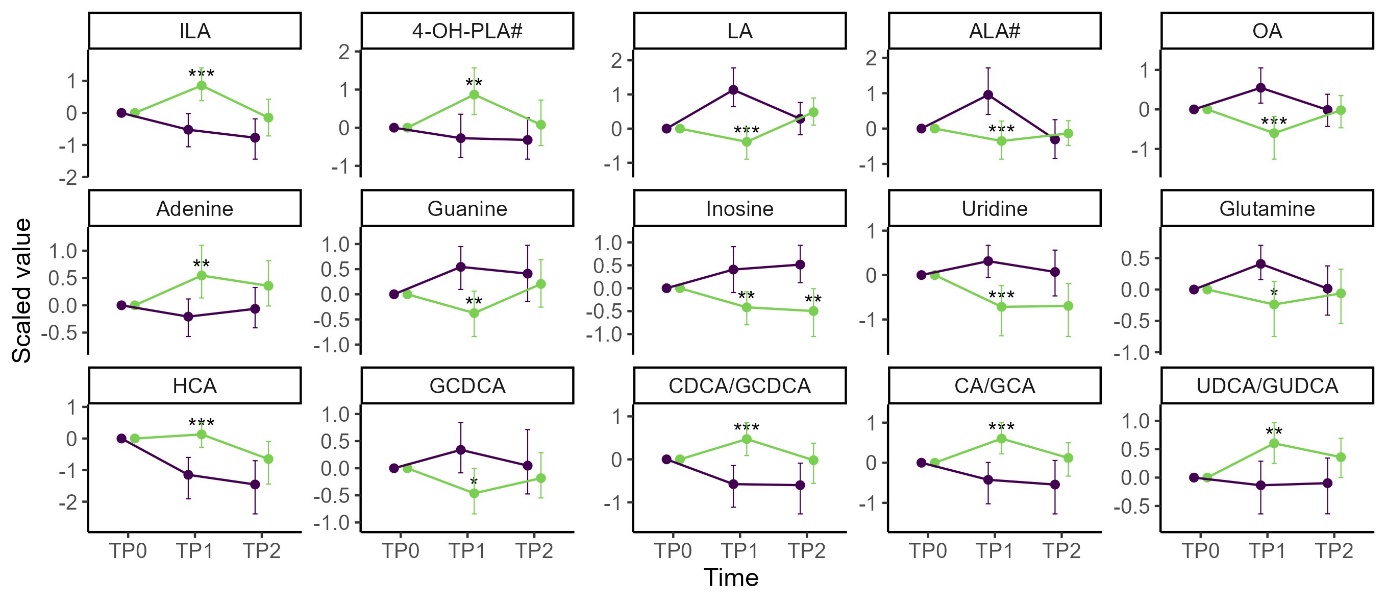
Figure S3. Marginal means estimated from the LMMs for AAF and AAF-S group. Only the metabolites for which an interaction coefficient was found significant are plotted. The response has been scaled. The q-values are based on/denote the significant between-group change in the within-group change from baseline. q < 0.01 (***), q < 0.05 (**), q <0.1 (*)


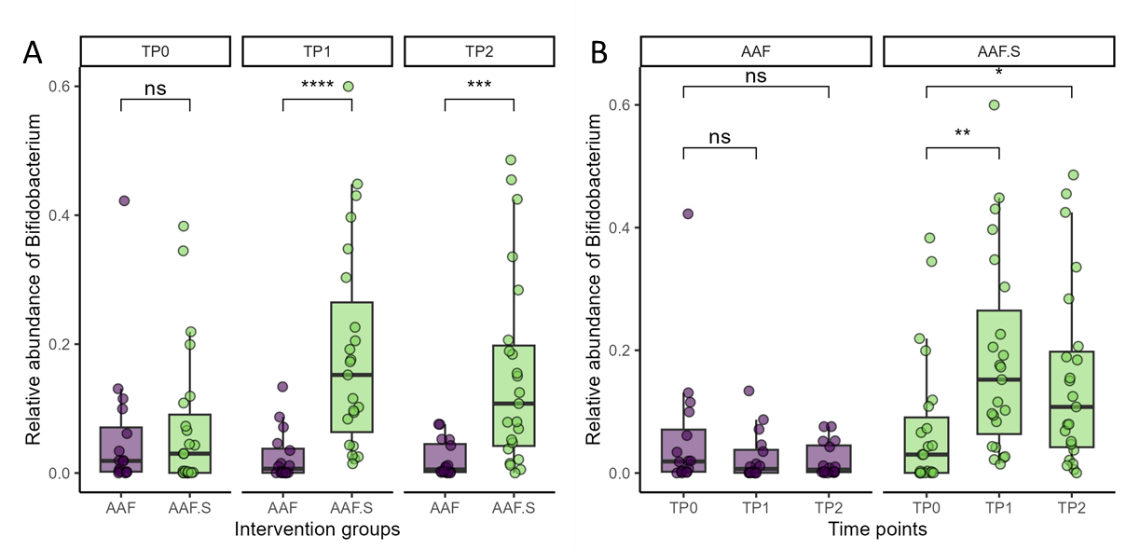


Figure S4. Relative abundance of *Bifidobacterium* comparisons between AAF and AAF-S groups at each time point (A), and between time points in each group (B). Statistical significance was evaluated with two-side unpaired t-tests; p > 0.05 (ns), p ≤ 0.05 (*), p ≤ 0.01 (**), p ≤ 0.001 (***), p ≤ 0.0001 (****).


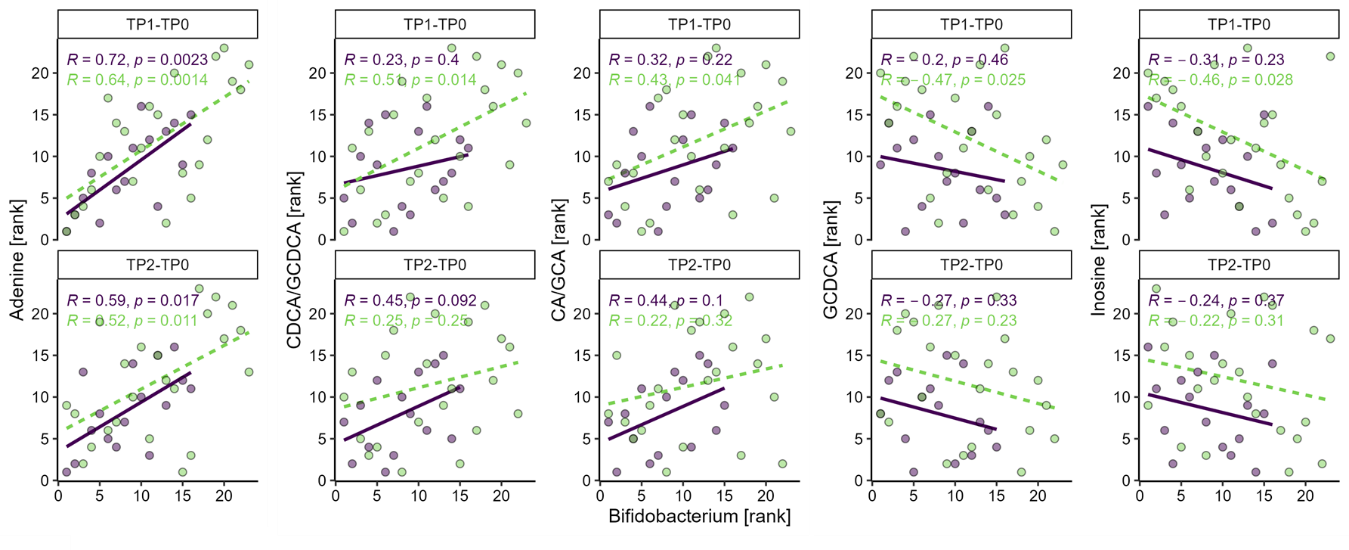


Figure S5. Spearman’s rank correlations between the changes in *Bifidobacterium* and adenine, CDCA/GCDCA, CA/GCA. GCDCA, inosine in AAF (purple solid line) and AAF-S (green dashed line) groups from baseline to TP1 (TP1-TP0) and TP2 (TP2-TP0). The rank of the changes in metabolite response and relative abundance of *Bifidobacterium* within each group were used for plotting.
